# Extracts from the Proceedings of the Buffalo Medical Association

**Published:** 1859-10

**Authors:** W. H. Butler

**Affiliations:** Secretary


ART. IV.—*Extracts from the Proceedings of the Buffalo Medical Association. August Meeting.* Reported by W. H. BUTLER, M. D., Secretary.

DR. ROCHESTER inquired if members had recently met with cases of diphtheritis. He had received letters from Drs. Hanks and Dunn, making inquiries if this disease prevailed associated with symptoms of croup. Dr. R. had seen two cases, which he regarded as scarlet fever with great inflammation of the fauces. One case terminated in gangrene and death. He had heard reports of such a disease in various parts of the State, but not previously as occurring in Buffalo. It was a question much discussed, if diphtheritic inflammation was usually connected or associated with scarlet fever.

DR. WILCOX gave some of the particulars of one case, of which he had the care, which had been seen, and was one of those referred to by Dr. Rochester. The child had attempted to swallow a toy-plate the day before, and the sore throat was thought to have been caused by the injury. Soon, diphtheritic effusion formed about the throat, and the child appeared as if sick with croup. Early and rapid prostration was a marked symptom. The treatment consisted of a few doses of hydrarg. cum creta, and Dover's powder.

DR. CRONYN made inquiry for the experience of members in the use of tinct. of cantharides and nitric acid in whooping-cough. He had found both equally efficacious—giving the acid preference in complicated cases.

He had treated about sixty cases giving the following :—

℞ Tinct. cinchonæ, ℥ iv.

— cantharidis, ʒ iij.

— camph. comp., ℥ ss.

Misce. Teaspoonful three times daily in a tablespoonful of flax-seed infusion.

In every case the severe paroxysms of cough had disappeared in ten days. He quoted Dr. Haynes, from *London Lancet*, that whooping-cough was as certainly cured with cantharides, as chlorosis with iron. He maintained that it was the cantharides which was mostly curative, since stranguiy seemed necessary to the cure. The medicine is to be continued until this effect is produced ; afterwards give it in small doses. This has been called the abortive treatment of Dr. Haynes.

DR. MINER had regarded medicine in the case of whooping-cough as of little avail ; he had recently consulted many of his medical friends as to their views of its treatment, having had a severe case in his own family.

Had heard no one pretend to knowledge of any remedy which was to be relied upon to abbreviate the time, or remove the cough; and with care for the general health, with anodynes when necessary, he had come to prescribe with great confidence *six weeks;* believing that, like many other diseases, it was self-limited and not to be controlled by medication. He was very much obliged to Dr. Cronyn for the suggestion, and hoped experience would prove that cantharides was truly a specific for whooping-cough.

Dr. Hamilton replied to Dr. Miner, that he had not called all his medical friends, for he had not been consulted. He would have relied upon $\frac{1}{8}$-grain doses of quinia, and out-door life. He saw something new in cantharides, and had heard of blistering the neck, chest, &c. If cantharides did actually cure at all, it was by acting as a counter-irritant. He thought the cinchona may possibly have been the important curative agent, if indeed the combination be found efficacious.

Dr. Cronyn had formerly given quinia, but regarded the fact of strangury being necessary before relief was obtained, as proof that it was the cantharides and not the quinia.

Dr. White has used tonics and other remedies; found ipecac. hive syrup, and all remedies of that class only to prolong the disease. He had known a blister placed upon the neck produce strangury, and not relieve the whooping-cough. Dr. White fears that the new treatment by Dr. Haynes will be found, like the numerous other plans which are constantly being claimed successful, to fail upon extended trial.

Dr. Rochester had heard mention of this plan; the complications are usually what we wish to treat in whooping-cough. The difficulty of diagnosis in the early stage constitutes one ground of fallacy, and may lead us to suppose we have cut short a case of whooping-cough, when really the case was one of catarrhal difficulty so apt to accompany the whooping-cough. He has often seen cases of severe paroxyms of coughing when there was afterwards found to be nothing of the spasmodic disease. He has tried belladonna, and obtained no abbreviation of disease more than with other anodynes.

Dr. Strong inquired if members had seen any thing of cholera? He had seen one very positive case, in a German, which yielded very readily to treatment; he had heard of other cases.

Dr. Treat had seen three cases of cholera morbus in the same neighborhood. One case came very near to collapse, it being four hours before reaction took place.

Dr. Miner inquired of Dr. Strong by what means he could decide that a case which yielded readily to treatment was very positive Asiatic cholera?

rather than severe cholera morbus. He objected to calling these cases occurring when nothing of the sort is prevailing here or elsewhere, cholera. He spoke of the occurrence of sporadic cholera as doubtful, or, if settled, as not best to report such cases to the public in that form, as it is difficult to make them believe in its non-contagion.

Dr. Strong replied that the Association decided in 1849 what is cholera, viz.: Those cases with rice or starch water stools shall be reported as cholera. After giving the symptoms, Dr. Strong did not hesitate to call this case cholera, saying, "We have more or less cases every year."

Dr. Hamilton wished to have it regarded as cholerine. He thought Dr. Miner had not seen rice water stools previous to the first visitation of cholera. Since that time, cases of that sort may be regarded as cholerine, if not positive Asiatic cholera; the disease has changed its type somewhat since that period.

Dr. Rochester wished to have these cases called cholera; and wants the people to know that the disease does prevail sporadically; he regards the difference between it and cholera morbus very manifest. Pain in the bowels in cholera morbus distinguishes it from cholera, which is almost always painless.

Dr. Treat objected to the distinction; contending that cholera is often attended with severe pain.

Dr. Miner could readily see that we were gaining nothing towards settling this question, which the Association had, according to Dr. Strong, done so conclusively in 1849. Rice-water stools do not belong exclusively to cholera, or fully distinguish it. Pain in the abdomen is not always absent in true cholera. Though Dr. Rochester regards the distinction very manifest, yet he is inclined to the belief that it is not so manifest in actual practice as in theory, especially in absence of any epidemic. For himself, he should prefer to call a case such as yielded readily to treatment, occurring alone, even though presenting the symptoms of cholera, cholera morbus; or, with Dr. Hamilton, cholerine, which word has been made to include all looseness of the bowels from whatever cause.

PINS AND NEEDLES PASSED THROUGH THE DIGESTIVE SYSTEM.

Dr. Storck reported the following case of pin-swallowing:—

On the 11th of July, in the morning, a girl 12½ years old, of healthy, robust appearance, step-daughter of Peter Maurice, on Genesee street, was brought into my office by her mother. The girl said she had a pin sticking in her throat, which she had swallowed a few minutes before. She complained of pricking pain far down in the œsophagus; which was severest in the act of swallowing.

I first introduced the throat forceps, carrying them far down into the throat, making efforts to feel and withdraw the pin; but these attempts were unsuccessful. I then introduced a small sponge attached to a curved whalebone, pushing it gradually down into the cardiac orifice of the stomach, and withdrawing the same again slowly, with the intention of removing the pin from its place and carrying it along with the sponge. I failed also in this; the pin did not appear, but the face of the girl brightened up; she said, "That pricking pain in my throat is gone, when I swallow." She felt considerably relieved. A piece of soft bread which was given to her, she swallowed without any difficulty. I concluded that in passing my sponge down into the œsophagus, I must have removed the pin from its position, and it passed into the stomach. I ordered some sweet-oil to be swallowed, and some farinaceous food to be given to her during the day.

In the evening I was called to see the patient. She suffered considerable colicy pain in her bowels; her abdomen was tympanitic, and tender to the touch. Her extremities were covered by a cold perspiration, and she was in great agitation. I ordered some castor-oil to be given to move her bowels, without regard to the pin, and left a dose of morphine.

Next morning, the mother of the child brought *seven pins* and *four* needles, of different sizes, into my office, stating that they had just passed from the child when she had a passage from her bowels, and that she had taken them herself out of the feces. The girl felt relieved during the day, but was again attacked in the evening with the same symptoms. She had used the same articles as food as she did the day before, viz., mashed potatoes, rice-pudding, etc. I merely ordered some sweet-oil to be swallowed, and also an enema of the same to be given per anum. During the night she had another passage from her bowels, and passed off *eight* more pins and *one* needle. In the afternoon, *four* more pins came away in the same manner. In the whole, *nineteen* pins and *five* needles. They all had a black appearance. The girl had suffered occasional colicy pain, during these four days; was greatly agitated, and would sometimes scream so loud that she could be heard in the neighboring houses. Her bowels were at times bloated, tender to the touch, and hard; but there were no marked constitutional symptoms; the girl would be up sometimes half a day walking about, without complaining at all.

A strict inquiry was then made, and the girl closely examined, to ascertain how she came to swallow so many pins and needles. Until then, she had denied ever having swallowed any but one, which got stuck fast in her throat. At last she acknowledged to the family that she had commenced swallowing pins and needles about the middle of April (after Easter), at the time when her parents intended to put her out to work. She had said about that time to another girl, that she wished she could get sick, so that she would not be obliged to leave home and work out. For this reason, she said, she had swallowed the pins.

The parents say that the girl had complained for some time back of pain in her bowels, and sometimes would not eat for a whole day. She went into service once, but was soon sent back again.

The girl, after these pins had passed, soon recovered, and enjoys now perfectly good health.

Prof. Hamilton said, that as to the treatment, he could see nothing in it to criticise. If, as was stated, the pin were pushed into the stomach during the attempt made to extract it, and if the castor-oil were given to relieve pain and tympanitis, without reference to the pin, then this also was proper. Indeed, there is plenty of authority for giving castor-oil for the purpose of expelling pins; yet this is not his practice.

As to the question of the probability of the occurrence, Dr. H. remarked that, without reference to any discussion which may have taken place outside of this hall, he saw no improbability in the statements which had been made. We certainly could not deny the possibility of such an occurrence; and the testimony was in this case sufficient to leave no ground for reasonable doubt. If it was rare to hear of examples in which patients had passed twenty-four pins, it was also rare to hear of cases in which persons had swallowed this number. Patients generally swallow them by accident, and then only one at a time; and these, as every practitioner knows, are generally passed in a few days. To pass is the rule, and to penetrate the walls of the abdomen is the exception.*

It was not known but that this patient had swallowed a hundred pins, and that they had been passing occasionally for several weeks; and that this sudden accumulation had been pressed forward by the castor-oil; these twenty-four may be but a fraction of the whole number. Now, if she has swallowed one hundred, certainly it is not strange that she would pass twenty-four.

Dr. White regrets any controversy, especially in the public papers. It is true, many cases of the kind are reported where physicians and friends are imposed upon. He agrees with Dr. Hamilton that there is nothing improbable in this case, and that it is well and satisfactorily sustained by the proofs. Dr. Delamater used to say, "Give yourself no trouble about such things said to be in the throat. If they pass down, they will pass through." Dr. Storck's treatment was entirely proper. Pins, needles, and other substances do often pass the alimentary canal; there can be no dis-

* In the "Copenhagen pin case," 400 pins came out through the body, and not one passed the rectum; but this patient had a stricture, which prevented the passage.

pute about it. They may, and probably often do, cause peritonitis, perforation, etc.; but they generally pass with the stools.

I regard it as due ourselves, to receive this report as very satisfactory and well authenticated. For himself, he does not regard giving cathartics as necessary when such substances have been swallowed. If the cathartic were administered for the purpose of relieving the pain, etc., it was very proper; with any other view, it might not have been admissible.

Dr. Treat regards the probability of such a case diminished by the number passed, or said to have passed, and that they did not sooner appear.

Dr. Hamilton regards that as casting no doubt upon so well authenticated a case. She may have swallowed—as he stated—more than reported, and passed a great number before attention was called to it.

Dr. Cronyn knew of one case where pins had passed the bowels, and one passed out at the side; another was distinctly felt and cut for by the surgeon, but not obtained.

Dr. White moved that the report of Dr. Storck be received and placed on file; seconded by Prof. Hamilton, and carried.*

### Proceedings of the September Meeting.

Dr. White reported a case, occurring in the practice of Dr. Newell, of the removal of a fibro-cartilaginous tumor from the posterior wall of the pharynx, in a child six years of age. The tumor was congenital. It caused violent paroxysms of coughing, suffocation, and occasionally asphyxia. Dr. White seized the tumor with the long forceps, and removed it by torsion. No hemorrhage followed, and the child is thriving well since the operation. The tumor is exceedingly firm, and had hardly grown at all since the birth of the child.

Dr. White reported a case of removal, in two instances, of the lower segment of the uterus; the former operation occurring the 2d of July last, the latter last Wednesday. In both cases, the operation was for the removal of cauliflower excrescences. He made use of the ecraseur. No hemorrhage followed the operation. He thinks it perfectly safe to remove

---

* In our Buffalo letter, which appeared in the last number, the case reported by Dr. Storck was mentioned; and also the reader will recollect that allusion was made to a discussion which had been carried on in two of the German papers of the city of Buffalo, in regard to the possibility of such a case. It is unnecessary to inform a medical man that cases are on record where a large number of pins and needles have been swallowed and passed per rectum. The case reported by Dr. Storck is unusual and of great interest. It does not appear that he examined the feces himself; but we are of course compelled often to trust to the representations of patients; and as such cases are not very unusual, and as all the other circumstances go to corroborate the assertions of the patient, this case must be regarded as authentic.—Editor.

the neck of the uterus in this manner. The disease is liable to return, as it is no doubt malignant. He was sorry he had not the specimen with him; but had left it with Dr. Lothrop for microscopic examination. These cases are very rare. Rokitansky mentions having never seen a morbid specimen of the kind.

Dr. Hamilton presented a specimen of false membrane of the trachea and bronchi, which he had received from a physician in Medina. There had been loss of voice for several years. Upon removal of the false membrane, voice and health were restored.

Dr. Hamilton is of the opinion that a person during sleep cannot be anæsthetized by chloroform. He recently tried the experiment, placing chloroform to the nostrils of a child. It at once turned away its head; again applying it, the child again turned away; the third trial, the child lifted up its head, pressed away the handkerchief, and awoke. This question is important in a medico-legal point. He hopes experiments will be made, and that others will pursue the inquiry; but is thoroughly convinced the effect cannot be produced, unless the person be drugged, or is in an unnatural sleep.

Dr. Cronyn confirmed Dr. Hamilton's views, by the relation of a case of a child asleep when anæsthesia was attempted, but could not be induced.

Dr. Rochester remarked that he had administered chloroform to a child forcibly held, when it instantly came under its influence, almost in a moment; so that he was alarmed, thinking he had, perhaps, administered too much. He thought this, however, an exceptional case.

Dr. White was happy the question had been raised, and was glad Dr. Hamilton had taken the initiative. He should be extremely cautious in coming to conclusions. Adults, being less susceptible to impressions, might be brought under the influence of anæsthesia, when a child would not. The fatigues of journeying in a railroad car might contribute to the susceptibility to anæsthesia. He would not commit himself; was skeptical, although inclined to the views of Dr. Hamilton. Unless a person was more profoundly asleep than usual, can scarcely conceive how anæsthesia can be induced.

Dr. Wyckoff remarked that his observations did not accord with those expressed by the other gentlemen. He entertained opposite views from them. If chloroform was held a distance from the nose, gradually accustoming the nostrils to it, a person, he thought, could be put to sleep. He had applied it to the nostrils of one who had delirium tremens, who swore against it; but he gradually administered it, and in the course of three quarters of an hour the patient was brought fully under its influence, which was kept up for seven hours.

Dr. White asked if, when inhaled, chloroform would not render obtuse the olfactory nerves, and anæsthetize the Schneiderian membrane?

Dr. Hamilton replied, such would probably be the case.

Dr. Wilcox asked if temperature, or seasons, would not produce some modifying effect, by retarding or facilitating evaporization?

Dr. Miner thought local anæsthesia, as recommended by Dr. White, to the uterus, &c., depended wholly upon the stimulating properties of chloroform.

Dr. White would not attempt to enlighten Dr. Miner by an explanation of its rationale; but knows that the vapor of chloroform passed up into the uterus through a vaginal tube, will relieve pain in five minutes. It should be repeated several times a day. When used endermically, much less effect must be produced than when applied to a mucous membrane.

Dr. White related a case of a patient, temporarily relieved of uterine pain produced by cancerous disease. Gas was generated by the union of carb. soda with an acid, and the fumes allowed to escape from a glass jar, through a vaginal tube into the uterus. This was resorted to when opiates had ceased to afford relief, or did not act. It may be owing to the stimulating effects of chloroform that anæsthesia is produced; the same effects may also be produced by ether.

Dr. Rochester thought if there was not actual contact of chloroform with the surface of a mucous membrane, there would be slight irritation produced. He had filled the bowl of a clay pipe with cotton saturated with chloroform, and blown the vapor through the stem into the ear to relieve pain of that organ.

Dr. Hamilton remarked that he could appreciate Dr. Miner's scepticism. Both laudanum and chloroform have been used when no local effect has been produced. Belladonna, when applied to the eye, produces dilatation of the pupil; which cannot be attributed to its stimulant properties, but to absorption. No doubt its effects have been greatly exaggerated; but when absorbed into the veins, its local effect is then produced.

Dr. Cronyn observed the almost instantaneous relief when chloroform was locally applied for toothache.

Dr. Hamilton thought the relief was wholly due to absorption.

Dr. Miner is of opinion that no more anæsthesia can be produced by the local application of chloroform than by the stimulant effect of camphor.

Dr. White thought *that* no proof of the want of anæsthetic power of chloroform.

Dr. Miner had seen a painful hand dipped into vapor of chloroform, without any relief whatever.

Dr. White thought that did not show that the Schneiderian membrane would not become anæsthetized, as the mucous membrane must be more susceptible than the outside covering.

Dr. Rogers thought both theories might be true in a degree. He saw no difficulty in reconciling both with the results produced.

Dr. Hamilton was of opinion that a robbery produced on the theory of the administration of chloroform, admitted of too many conditions to be reconciled to his notions of truth: lack of susceptibility, cautious approach, or rapid approach, which would arouse the person; and presuming the robbery to be attempted in the cars, would not others soon know it? One poor fellow was now in prison for a robbery in the cars. He does not doubt the robbery, but does not believe he put the person under the influence of chloroform to accomplish it.

Dr. Wyckoff reported two cases of whooping-cough relieved by cantharides. Children had been whooping one week, which yielded to the cantharides in ten days. In another case, that of his own child, the disease was decidedly cut short by the use of the hydrocyanic acid. He discontinued its use for three or four days, when the cough increased, and got worse; again using the medicine, and again relieved the patient.

Dr. Gould presented the Society a specimen of tape-worm. Four ounces of emulsion of pumpkin-seed was used without success. He attributes the expulsion of the worm to a mixture of turpentine with castor-oil—one tablespoonful of the former, with two tablespoonfuls of the latter.

